# Comparative transcriptomic and evolutionary analysis of *FAD*-like genes of *Brassica* species revealed their role in fatty acid biosynthesis and stress tolerance

**DOI:** 10.1186/s12870-023-04232-9

**Published:** 2023-05-12

**Authors:** Nabeel Shaheen, Uzair Muhammad Khan, Ayesha Farooq, Ummul Buneen Zafar, Sultan Habibullah Khan, Shakeel Ahmad, Muhammad Tehseen Azhar, Rana Muhammad Atif, Iqrar Ahmad Rana, Hyojin Seo

**Affiliations:** 1 Department of Plant Breeding and Genetics, University of Agriculture, Faisalabad, 38000 Pakistan; 2 Center for Advanced Studies in Agriculture and Food security, University of Agriculture, Faisalabad, 38000 Pakistan; 3 Seed Center and Plant Genetic Resources Bank, Ministry of Environment, Water & Agriculture, Riyadh, 14712 Saudi Arabia; 4 Center of Agricultural Biochemistry and Biotechnology, University of Agriculture Faisalabad, Faisalabad, 38000 Pakistan; 5 School of Agriculture Sciences, Zhengzhou University, Zhengzhou, 450000 China; 6 Precision Agriculture and Analytics Lab, National Center in Big Data and Cloud Computing (NCBC), University of Agriculture Faisalabad, Faisalabad, Pakistan; 7 Korea Soybean Research Institute, Jinju, 52840 Korea

**Keywords:** Fatty acid desaturase, *B. carinata*, Genome wide identification, Phylogenetic, Transcriptomic data analysis

## Abstract

**Background:**

*Fatty acid desaturases* (*FADs*) are involved in regulating plant fatty acid composition by adding double bonds to growing hydrocarbon chain. Apart from regulating fatty acid composition *FADs* are of great importance, and are involved in stress responsiveness, plant development, and defense mechanisms. *FADs* have been extensively studied in crop plants, and are broadly classed into soluble and non-soluble fatty acids. However, *FADs* have not yet been characterized in *Brassica carinata* and its progenitors.

**Results:**

Here we have performed comparative genome-wide identification of *FADs* and have identified 131 soluble and 28 non-soluble *FADs* in allotetraploid *B. carinata* and its diploid parents. Most soluble FAD proteins are predicted to be resided in endomembrane system, whereas FAB proteins were found to be localized in chloroplast. Phylogenetic analysis classed the soluble and non-soluble FAD proteins into seven and four clusters, respectively. Positive type of selection seemed to be dominant in both *FADs* suggesting the impact of evolution on these gene families. Upstream regions of both *FADs* were enriched in stress related *cis*-regulatory elements and among them ABRE type of elements were in abundance. Comparative transcriptomic data analysis output highlighted that *FADs* expression reduced gradually in mature seed and embryonic tissues. Moreover, under heat stress during seed and embryo development seven genes remained up-regulated regardless of external stress. Three *FADs* were only induced under elevated temperature whereas five genes were upregulated under *Xanthomonas campestris* stress suggesting their involvement in abiotic and biotic stress response.

**Conclusions:**

The current study provides insights into the evolution of *FADs* and their role in *B. carinata* under stress conditions. Moreover, the functional characterization of stress-related genes would exploit their utilization in future breeding programs of *B.* carinata and its progenitors.

**Supplementary Information:**

The online version contains supplementary material available at 10.1186/s12870-023-04232-9.

## Introduction

*Brassicaceae* family is comprised of 372 genera and 4006 species and members of this family are sources of fodder, oil, food, condiments, and biofuel. *B. carinata* (BBCC), an allotetraploid (2n = 4x = 34) came into birth after the natural hybridization between *B. oleracea* (CC) (2n = 2x = 18) and *B. nigra* (BB) (2n = 2x = 16) about 0.47 million years ago (MYA). The allotetraploid *B. carinata* is known for its climate resilience characteristics like heat, drought, and lodging resistance. It also has resistance against biotic stresses as well [1]. *Brassicas* are third source of oilseed after palm and Soybean. The *B. carinata* is popular for industrial use due to higher Erusic acid (22:1) [2]. However, its diploid progenitors are being used for condiments and vegetables. Vegetable oils are crucial part of the human diet carrying important fatty acids [3]. They also serve as an important energy source and are major constituent of most biological membranes [4]. The acetyl-CoA provides raw material to synthesize fatty acids in plastids [5]. Production of C16 to C18 carbon fatty acids require 30 enzymatic reactions in the stroma of plastids [6]. Fatty acids are categorized into polyunsaturated, mono-unsaturated, and saturated ones. Unsaturated fatty acids constitute 75% of total fatty acids and the concentration of unsaturated fatty acids is higher in higher plants compared to other living matter. Moreover, their proportion in oil also determines the quality of edible oil [7, 8].

*FADs* converts the saturated fatty acids to unsaturated fatty acids by modifying single bonds to double bonds at specific sites [9]. *FADs* are broadly categorized into soluble and non-soluble *FADs* [10]. Moreover, this gene family is further divided into five sub-gene families; *ADS* (*Acyl-CoA-desaturase*), *DES* (*Sphingolipid Δ-4 desaturase*), *FAB* (*Δ-9 stearoyl-ACP desaturase*), *FAD*, and *SLD* (*Sphingolipid Δ-8 desaturase*) [11]. Among soluble desaturases, only *FAB2* have been identified in plants [12] which catalyzes the conversion of stearic acid (18:0) to oleic acid (18:1) by incorporating a double bond at Δ9 position [11]. Non soluble *FADs* are further classified into Omega-3 (ω3﻿) desaturases (*FAD3*/*FAD7*/*FAD8*), and Omega-6 (ω6) desaturases (*FAD2*/*FAD6*, and *FAD4*). The ω3, ω6, and *FAD4* are involved in regulation of linoleic acid (18:2), linolenic acid (18:3), and palmitoleic acid (16:1) biosynthesis respectively [13]. The *FAB2*, *FAD4*, *FAD6*, *FAD7*, and *FAD8* are involved in lipid desaturation in plastids whereas *FAD2* and *FAD3* performed the same function in the endoplasmic reticulum [14, 15]. Previous reports suggested that *FADs* from the same family/subfamily possess conserved amino acid sequences. For instance, two (D/ EXXH) and three (H(X_3 − 4_ H/H(X)_2−3_HH/H/Q(X)_2−3_HH)) histidine motifs are conserved in *FAB2s* and membrane-bound *FADs* respectively [16].

Although *FADs* are mainly involved in determining edible oil quality but, their role in abiotic stress responses has also been reported. According to Iba [17] concentration of unsaturated fatty acids determines the plant tolerance level against heat and cold stresses. Moreover, unsaturated fatty acids are also required to maintain cell membrane fluidity during adverse environmental conditions [18]. For instance, expression of At*FAD8* gene abruptly increased under cold stress while expression of At*FAD6* and At*FAD8* genes induced at seedlings level against osmotic and salinity stresses [18–21]. Similarly, the *GmFAD3* and *GmFAD7* genes were found to be abundantly express under low temperature [22]. The *LeFAD7* gene has been found to be negatively correlated with heat stress [23]. Moreover, heterologous expression of *AtFAD3*/*AtFAD8* genes in tobbacco showed enhanced osmotic tolerance [24].

The availability of whole genome sequence of crop plants provides the opportunity to study any gene family. Consequently, the *FAD* gene family members have been studied in *Arabidopsis* [25], *Cucumis sativus* [14], *Glycine max* [26], *Linum usitatissimum* [27], *Medicago trancula* [28], *Musa* spp. [29], *Cicer arietinum* [13], *Camelina sativa* [30], *Gossypium hirsutum* [18], *Medicago sativa* [31], *B. napus* [11] and *B. Juncea* [16]. However, *FADs* have not been yet studied in *B. carinata* and its progenitors. Therefore, in this study, we have performed gene identification, phylogenetics, chromosomal localization, promoter and gene structure analyses in *B. carinata* and its progenitors. In addition, the role of *FAD* genes under normal and stressed conditions have also been explored using available transcriptomic data of different plant tissues. The findings of gene divergence analysis, and synteny analysis would certainly give insight into evolution of this gene family members.

## Results

### Gene identification and phylogenetics of *FAD* gene family

**Fig. 1:**
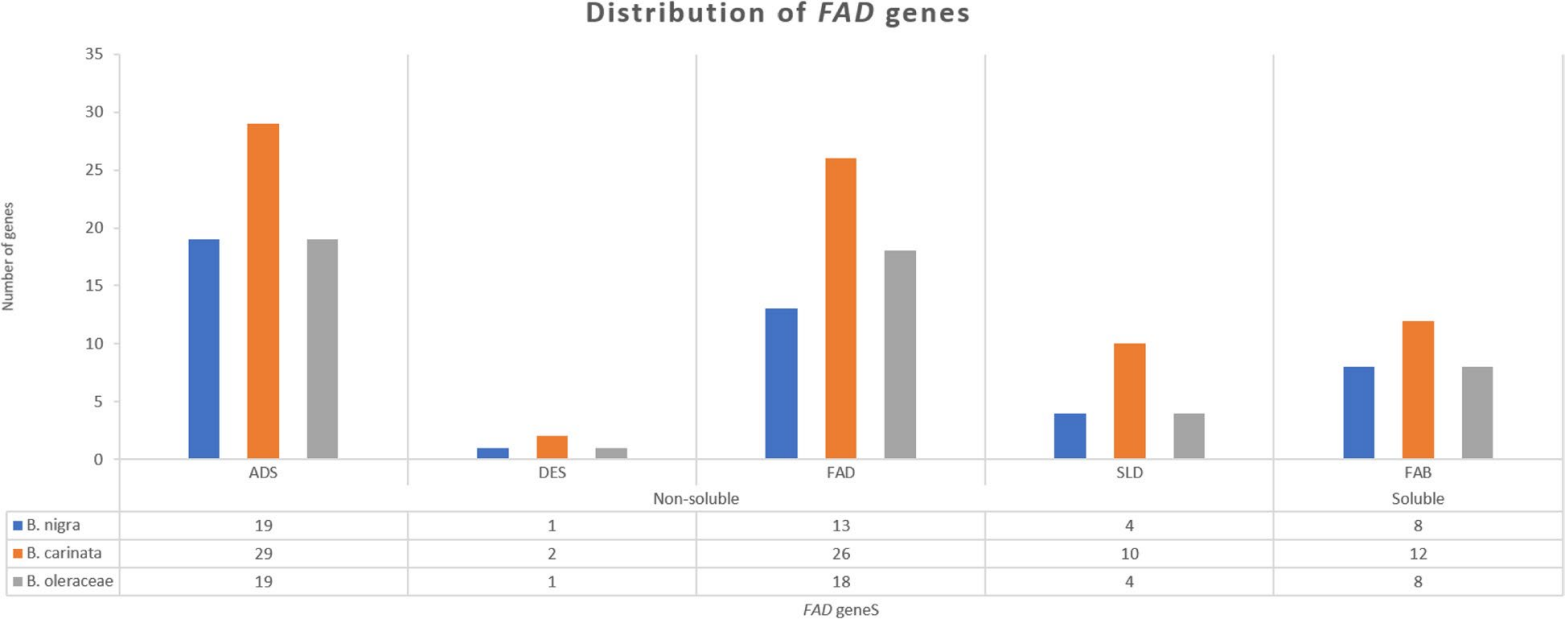
Distribution of soluble and non-soluble *FADs *in diploid and tetraploid *Brassica* genomes

Genomes of *B. carinata, B. nigra*, and *B. oleracea* were searched against the query sequences of FAD proteins of *A. thaliana* to know the distribution of *FAD* gene family members in *Brassica* genomes. The conserved domain database (CDD) was used to check the conserve domains of each protein and genes having the conserve domain were used in further analysis. Finally, 67 *ADS*, 57 *FAD*, 28 *FAB*, 18 *SLD* and 04 *DES* genes were shortlisted after confirming their conserved domains (Fig. 1). The shortlisted genes were grouped into five sub-gene families (*SLD*, *ADS*, *DES*, *FAD*, and *FAB*) as suggested in previous literature of *Brassicas* [11, 16] (Table S1). The deleted incomplete domain length could be the result of evolution as genes might lost their part during evolution and considered as pseudo-genes [32]. Gene length varied between and within gene families and *BolADS15* was the lengthiest gene followed by *BcaFAD3.1 A*, *BcaFAB2.2.1 A*, *BcaDES02*, and *BolSLD1.2* genes (Table S1). Moreover, other gene related information like protein length, isoelectric point (*pI*) and coding sequence length are listed in Table S1. The identified genes were renamed according to their chromosomal position i.e., *BniB01g012460.2 N.1* is the first gene located on B1 chromosome of *B. nigra* and renamed as *BniADS01*. Prediction of subcellular localization concluded that most membrane bound desaturases resided in endomembrane system, comparing to *FABs* that were predicted to be localized in chloroplast (Table S1). Since endomembrane system is comprised of complex membrane trafficking network crucial in material exchange and transport, and it might be possible that genes located within this system could be involved in transportation [33].

### Phylogenetics and motif analysis of FAD proteins of *Brassicas*

Reliable gene phylogeny could help to determine the function and structure of uncharacterized protein [34, 35]. Comparative phylogeny of membrane bound FADs (Figure S 1), and soluble FAB2s (Figure S 2) related proteins were carried out separately due to higher dissimilarities between them.

The phylogenetics of FAD proteins clustered into seven clusters. Cluster VII contained 76 proteins followed by cluster IV and cluster VII carrying 27 and 17 proteins from *Brassicas*, *O. sativa*, and *A. thaliana*, respectively. Cluster VII contained 25 orthologous gene pairs compared to cluster IV, cluster V and cluster III which had 06, 04, and 03 orthologous gene pairs, respectively. Cluster II was devoid of any orthologous gene pairs, and cluster I and cluster III each carried one orthologous gene pair. Moreover, it is interesting that each gene family from group I resided in separate clade  that might be the indication of their independent evolution (Figure S 1). FAB proteins were grouped into four groups and group III had five orthologous gene pairs followed by group IV, and group II which carried four and three orthologous gene pairs respectively. Interestingly group II and III had not any *O. sativa* protein (Figure S 2).

**Fig. 2:**
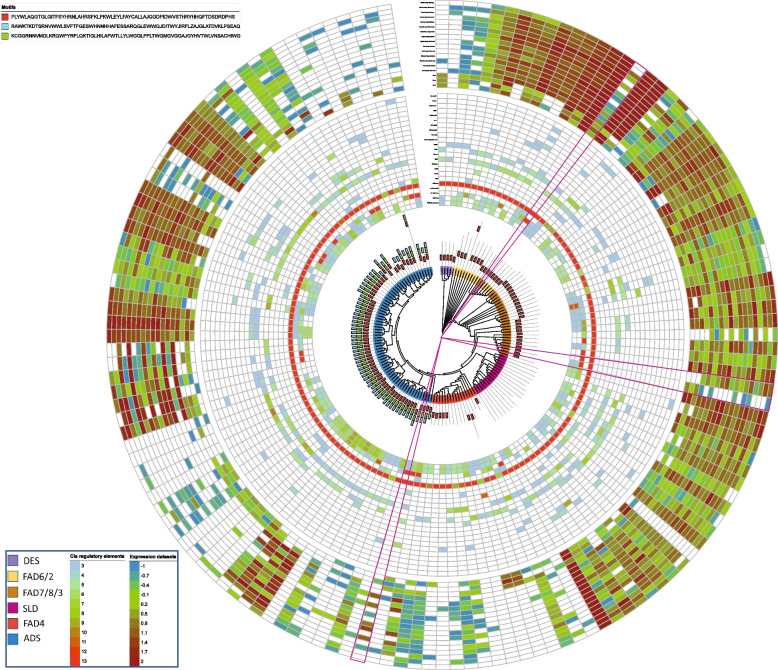
Phylogenetic, motif composition, cis-regulatory and expression analysis of non-soluble *FADs *in different tissues

**Fig. 3:**
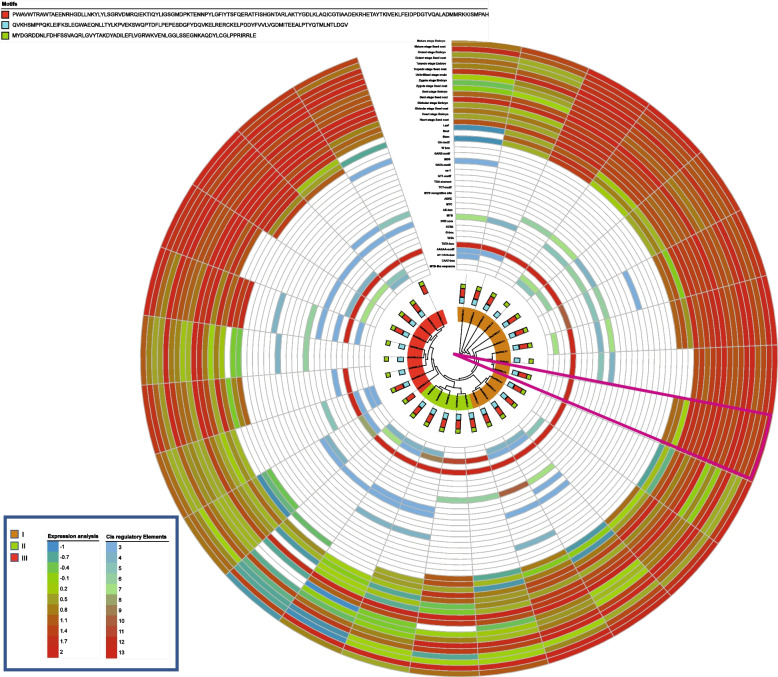
Phylogenetic, motif composition, cis-regulatory and expression analysis of *FAB2 *in different tissues

Furthermore, MEME web server was used to identify the diversity of motifs in protein sequences. Finally, three types of motifs were identified in both FAD and FAB proteins. Motif 1 was common in all non-soluble FAD proteins except proteins of group IV. Protein of group I, II, III, and V had only motif 1 while group VI had all three motifs. The BolADS19 protein showed unique motif composition (Fig. 2). Like FADs, FAB2 proteins also had three motifs and proteins of group I had all three motifs except BcaFAB2.3.3 and BcaFAB2.3.2 proteins. The BcaFAB2.2.4 A, BniFAB2.2.4 A, and BolFAB2.2.2 A from clade III carried motif 2 and motif3 compared to BcaFAB2.2.1 A which had motif 1 and motif 3 respectively (Fig. 3).

### Physical gene localization of *FAD* related genes

**Fig. 4:**
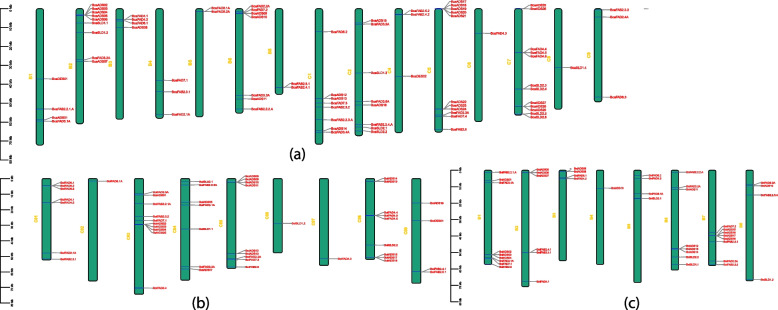
Chromosomal localization of soluble and non-soluble *FADs*

Genes were randomly and unevenly distributed on all chromosomes (chr). The *ADS* genes were localized in cluster form on both BB and CC chromosomes compared to *FAD4* genes which were found in cluster form in *B. carinata* (Fig. 4a) chromosomes only that could be the indication of QTL formation. For *ADS* genes, *B. nigra* chromosomes contained 23 genes and among these chrB01, chrB06, and chrB07 each contained 04 genes (Fig. 4c). Four chromosomes viz., chrB01, chrB04, chrB08, and chrB02, each had only 01 gene. Twenty genes were resided on CC chromosomes of *B. oleracea* and chrC05 carried highest number of genes (06) followed by chrC03, chrC08, chrC04, and chrC09 which contained 05, 05, 02, and 01 genes respectively (Fig. 4b). Four *DES* genes were localized on different chromosomes and each gene resided on separate chromosomes. For *FAB* genes, 05 and 06 genes were localized on BB chromosomes and CC chromosomes of *B. carinata* respectively. The chrB08, chrC01, and chrC04 each had 02 genes.

### Gene structure of *FAD* related genes of *B. carinata* and its parents

**Fig. 5:**
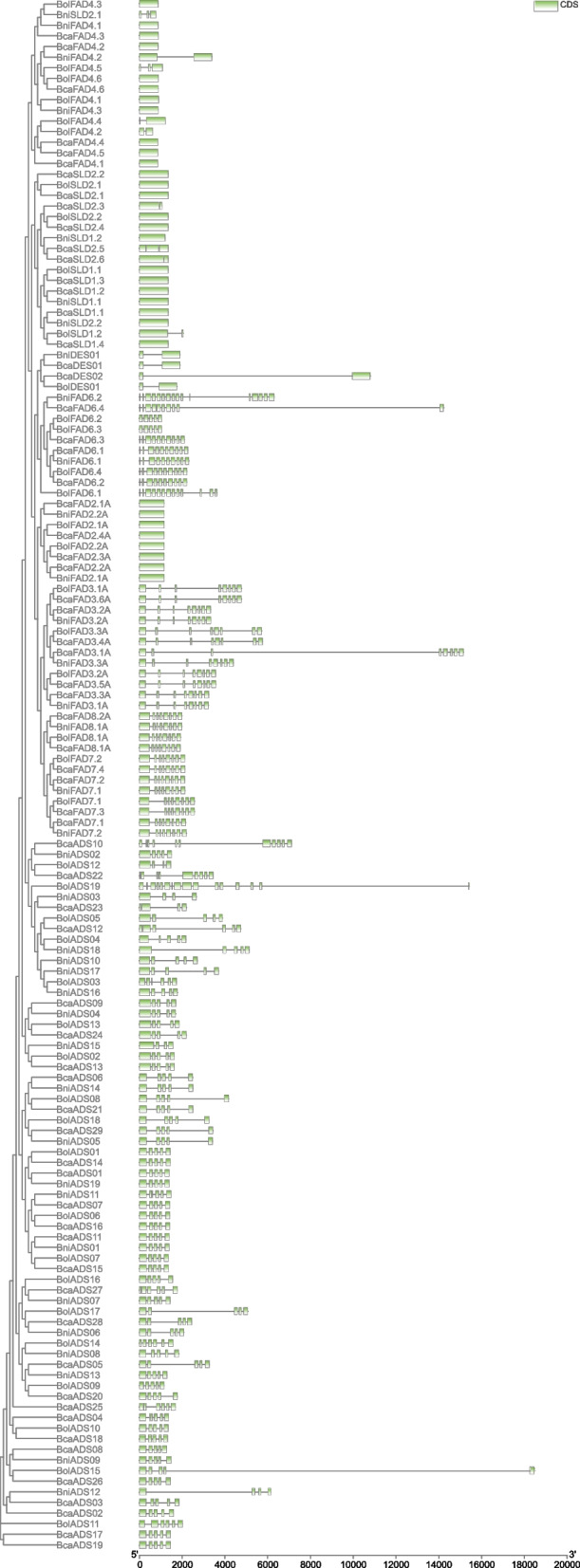
Gene structure of non-soluble *FAD *genes

Gene structure analysis provides insights into evolutionary pattern of gene family [36]. Gene structure was conserved within sub-gene families and varied between sub-gene families. The *DES* and *SLD* genes were intron less and remained conserved during evolution. Interestingly gene structure were highly conserved within *FAD3*, *FAD7*, and *FAD8* sub-gene family members. However, *BcaFAD3.1 A* showed variable intron distribution. The *FAD6* related genes were enriched in introns and *BcaFAD6.2* gene contained 15 introns. Moreover, genes carrying 9 introns constitute 60% of the gene family members (Fig. 5).

**Fig. 6:**
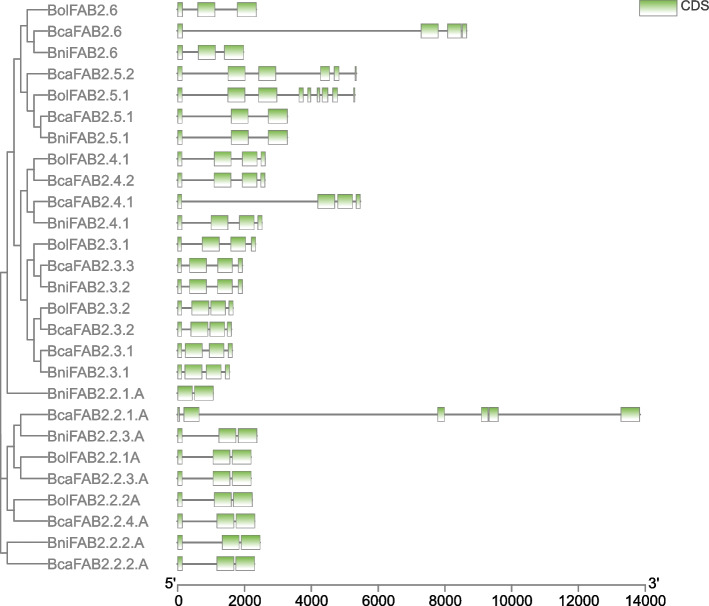
Gene structure of soluble *FAB2* genes

Out of total 67 *ADS* genes 53 had 04 introns and *BolADS19* showed highest intron number (15). Each *DES* genes contained 01 intron and *BcaDES02* gene showed variable intron/exon distribution compared to others. *FAB* genes have also showed conserved gene structure and genes with 02 and 03 introns shared similar intron and exon distribution (Fig. 6).

### Prediction of *Cis*-regulatory elements in upstream regions of *FAD* and *FAB* genes

Presence of *cis*- regulatory elements in upstream regions of gene greatly influence its function [36]. Initially, identified *cis* elements were classed into developmental, stress, light, and hormonal responsive as suggested in earlier literature [11]. Developmental type of elements include MYC, MYB-recognition, MYB-like sequences, HD-zip3, Myb, GATA-motif, CAAT-box, CAT-box whereas, GT-1-motif, AE-box, and TCT-motif placed in light responsive type of elements. Moreover, ABRE, STRE, as-1, GA-motif, G-box, DRE core, W-box and MBS belong to stress responsive class. Whereas, GARE-motif and TGA-motif were classed as hormonal responsive elements. Primarily, TATA-box a developmental type of elements were in abundance in all genes suggesting their role in developmental processes. Further, genes having abundunt TATA box elements got upregulated against biotic and abiotic stress responses (Fig. 2) [37]. Similarly, five genes viz., *BcaADS02*, *BcaFAD2.1 A*, *BcaFAD3.6 A*, *BniFAB2.3.2*, and *BolFAD3.1 A* had stress type of elements indicating their potential in stress responsiveness (Figs. 2 and 3 and Table S2). However, compared to other elements, ABRE were abundantly found in most *FADs* might be the indication of their involvement in abiotic stress responsiveness especially cold, drought and salinity [38–42] (Figs. 2 and 3).

### Gene divergence and syntenic analysis

Gene divergence is estimated through ka (Synonymous substitution)/ks (non-synonymous substitution) ratio, and this ratio is also used to predict the selection type [43]. If, Ka/ks remains > 1 it suggests positive selection [44], and equals to 1 or less than 1 indicates neutral [45] and negative type [46] of selection, respectively. The members of membrane bound *FADs* seemed to be go through extensive selection. Like, 12 and 19 orthologues gene pairs undergo positive and negative selection, respectively. Further, none of the genes showed neutral type of selection (Table S3). Compared to *FAD* genes, *FAB* seemed to have experienced lower selection pressure during evolution and only seven orthologous gene pairs depicted impact of evolution. Out of these seven pairs, three had experienced positive, three went through negative and only one pair had faced neutral selection (Table S3).

**Fig. 7:**
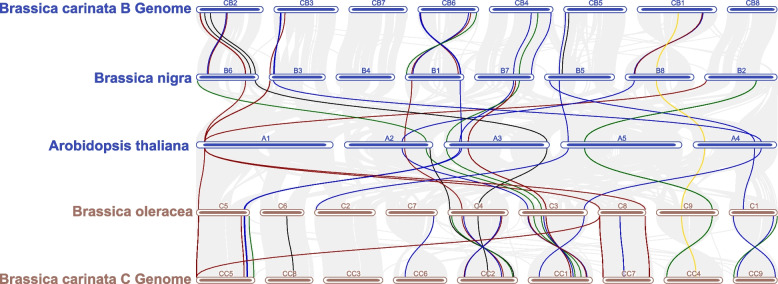
Syntenic analysis of soluble and non-soluble *FADs*

Moreover, Genomes evolution could be studied through doing multiple synteny along with model organism [47]. Chromosomes of B genome of *B. carinata* i.e., CB3, CB4, and CB5 shared collinear genes with *B. nigra* chromosomes viz., B3, B5, and B7 respectively. The CC5, CC6, CC7, and CC8 of *B. carinata* contributed maximum to C5, C6, C7, and C8 chromosomes of *B. oleracea*. Additionally, besides showing collinearity block multiple synteny also allows to explore the inversions and translocation within and between chromosomes. For instance, CB1, CB2, and CB6 showed inversions with B1, B2, and B8 chromosomes of *B. nigra*. Likewise, The CC chromosomes (CC2, CC4, and CC9) of *B. carinata* also have inversions with *B. oleracea* (C1, C3, and C4) chromosomes (Fig. 7).

Red lines showing *ADS* genes, whereas *DES*, *FADs*, *SLD*, and *FAB2s* genes are highlighted by yellow, blue, black, and green lines respectively.

### RNA-seq data analysis of *FAD* genes in vegetative and reproductive tissues

RNA-seq data of both vegetative and reproductive tissues were analyzed to quantify the gene expression in different tissue. In vegetative tissues (Leaf, root, and stem) only genes from allotetraploid *B. carinata* showed abundant gene expression (*BcaFAD4.1*, *BcaFAD7.1*, *BcaFAD2.1*, *BcaDES01*, *BcaDES02*, *BcaADS12*, *BcaADS20*, and *BcaADS27*). likewise, during seed development, and embryo development 08 genes of *B. carinata* (*BcaSLD1.3*, *BcaFAD2.2 A*, *BcaFAD2.3 A*, *BcaFAB2.2.1 A*, *BcaFAB2.3.1 A*, *BcaADS11*, *BcaADS14*, and *BcaADS24*), and 06 genes of *B. nigra* (*BniSLD2.1*, *BniFAD2.1 A*, *BniFAB2.3.1*, *BniFAB2.3.2*, *BniADS04*, and *BniADS19*) were found to be abundantly expressed. Moreover, abundant expression of these genes in mature seed tissues could be an indication of their long transcription cycle and involvement in seed development (Figs. 2 and 3, and Table S2). Some genes expressed abundantly in all stages, but their expression markedly reduced in mature embryo and seed coat tissues. These gene include nine from *B. carinata* (*BcaSLD1.1*, *BcaSLD1.4*, *BcaSLD2.5*, *BcaFAD2.1 A*, *BcaFAD8.1*, *BcaFAD8.2 A*, *BcaFAD6.2*, *BcaFAD7.3*, *BcaFAD6.4*) and nine from *B. nigra* (*BniSLD2.2*, *BniSLD1.1*, *BniSLD1.2*, *BniFAD4.1*, *BniFAD8.1 A*, *BniFAB2.2.3 A*, *BniADS08*, *BniADS12*, and *BniADS14*). Interestingly, none of the genes from *B. oleraceae* showed higher expression in vegetative and reproductive tissues (Table S2).

### *FAD* genes got upregulated under heat, and *X. campestris* stresses

**Fig. 8:**
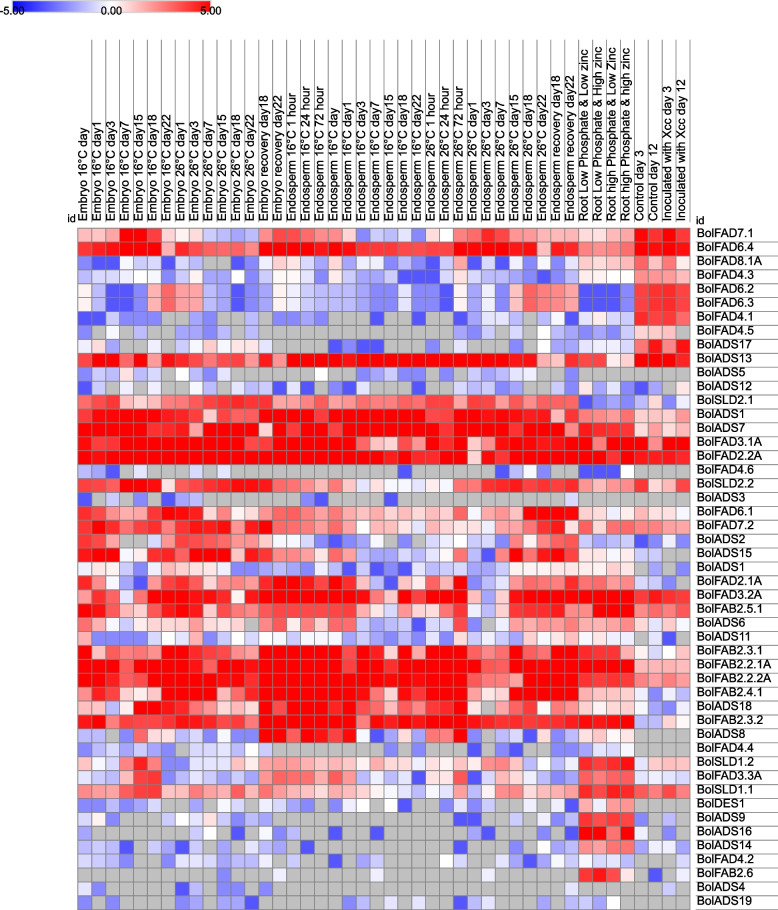
Expression analysis of soluble and non-soluble *FADs* under abiotic and biotic stresses

RNA sequencing under stressed conditions gives opportunities to identify potential genes that might trigger plant defense systems against stresses [48]. Four genes viz., *BolFAD6.4*, *BolADS13*, *BolFAD3.1 A*, and *BolFAD2.2* remained upregulated in all tissues regardless of temperature and day spans. Similarly, three genes (*BolSLD2.1*, *BolADS1*, and *FAD2.1 A*) were expressed at a higher rate in reproductive tissues (seed, endosperm, and embryo) only. These genes might have a role in fatty acid biosynthesis since majority of fatty acid biosynthesis occurs in seed tissues. Moreover, abundant expression of these genes under heat stress could also be an indication of their potential role in heat tolerance. In root tissues under phosphate and zinc stresses, six genes (*BolSLD1.2*, *BolSLD1.1*, *BolADS9*, *BolADS14*, *BolADS16*, *BolFAB2.6*) got upregulated. Among these six genes, *BolSLD1.1* is the only gene that is expressed under both biotic and abiotic stresses in vegetative tissues highlighting its involvement in producing defense-related proteins to cope with heat and pathogen stress. Apart from abiotic stress, the expression of *FADs* was also quantified under *X. campestris* inoculation. It has been observed that five genes viz., *BolFAD6.2*, *BolFAD4.3*, *BolFAD6.3*, *BolFAD4.1*, and *BolADS17* were upregulated under pathogen infection and could be further explored to confirm their resistance level against biotic stress (Fig. 8, and Table S4). Moreover, their functional characterization would further highlight their potential application in breeding climate-resilient *Brassicas*.

## Discussion

### Gene identification and their physio-chemical properties

Until now *FADs* have been identified in *Musa* spp. [29], *Cicer arietinum* [13], C*amelina sativa* [30], *Gossypium hirsutum* [18, 49], *Medicago sativa* [31], *Brassica* [16], and *Medicago trauncatula* [28]. Gene numbers varied from crop to crop, and we have identified 174 total *FAD* genes in *B. carinata* and its diploid progenitors [16]. The *FAD* genes are not following the genome sizes. For example, *FAD* genes are more in *A. thaliana* [25] compared to *G. hirsutum* [18], *T. aestivum* [50], and *B. juncea* [16] but equal to *Musa spp*. [29]. Moreover, *FAD* genes are less in number in *Juglans regia* [51], *Cucumis sativus* [14], and *O. sativa* [52] compared to *A. thaliana*. The same trend could also be observed in *B. carinata* and *B. napus* genomes where *FAD* genes are equal in both allotetraploids regardless of their genomes size. Further, identified genes could be classified as *FAB*, *SLD*, *ADS*, *DES*, and *FAD2*/ *FAD3*/ *FAD4*/ *FAD6*/ *FAD7*/ *FAD8* as reported in other *Brassica* species [16] and *T.aestivum* [50] but *C. arietinum* genome contains *FAD5* only [13]. Moreover, *O. sativa* and *Musa* spp. genomes lack *ADS* gene family implying that *ADS* came into birth after separation of monocots and dicots. During polyploidization *Brassica* genome might have lost *ADS, FAB*, and *FAD* genes as sum of genes of allotetraploid off-spring is less to the diploid progenitors genes (Fig. 1).

The FAD2 and FAD3 proteins were predicted to be localized in ER whereas FAD4, FAD6, and FAD7 proteins were predicted to be present in chloroplast thylakoid membrane, mitochondrial membrane, and chloroplast outer membrane accordingly as reported earlier in *T. aestivum*, *Musa* spp., *H. annus*, *L. usitatissimum*, and *T. cacao* genomes respectively [27, 29, 50, 53].

Gene structure is crucial in gene functioning and deviation in gene structure could provide insight into the evolution process [54]. The gene structure of *FAD* gene family is conserved across the species [11, 28, 29, 50, 52].

### Phylogenetics of FAD proteins

Protein sequences of both soluble and non-soluble FAD proteins were subjected to phylogenetics since signal-to noise ratio of protein sequences are more suitable to analyse gene families [55]. Phylogenetics of soluble and non-soluble were separately performed due to higher dissimilarities at protein sequence level. Like, *B. carinata* FAB proteins are also dissimilar in *C. arietinum* [13] and *B. napus* [11] but are alike enough to construct a combine phylogenetic tree in *Musa* spp. [29], *M. truncatula* [28], *M. sativa* [31], and *T. aestivum* [50]. Deviation of protein sequences in sub-family protein might be due to the significant impact of evolution and polyploidization of genomes. Moreover, phylogenetic clade formation also helps to determine the homology between protein sequences. For example, non-soluble proteins of cluster I contain AtDES1 suggesting its homology with adjacent proteins whereas members of cluster II and cluster V had sequence similarities with AthFAD6, and AthFAD4 proteins, respectively. AthFAD4 involved in palmitic acid (16: 0) synthesis [56] and AthFAD6 [57] introduces double bond to 16:3 and proteins of these clusters might have relevant functions.

Cluster III contains 04 AtFAD proteins viz., AthFAD2A, AthFAD3A, AthFAD7, and AthFAD8A whereas, cluster VI contains 10 AtADS proteins viz., AthADS01, AthADS02, AthADS03, AthADS04, AthADS05, AthADS06, AthADS07, AthADS08, AthADS09, AthADS76. Some of these protein have role in carbon chain modification whereas some are involved in conversion of the fatty acids. For instance, the AthFAD2A changes oleic acid to linoleic acid and AthFAD3 coverts it to linolenic acid. Similarly, AthFAD8 encodes trienoic fatty acid (16:2 and 18:2) which serves as substrate for the activity of AthFAD7 and further desaturation of these products takes place in chloroplast by AthFAD7 [19, 58]. Although FAD proteins are mainly involved in FA elongation. However, they also have potential role in stress responsiveness.

### Role of soluble and non-soluble *FAD* genes in biological processes

Exploring transcriptomic data under normal and stressed conditions helps to determine or to predict the gene’s function in biological processes [11]. Mainly, *FAD* genes are involved in pollen development [59], nodule and leaf development [60] and endosperm development [61]. The *BolFAD2* got up-regulated during seed and endosperm development but in soybean this gene predominantly expressed in vegetative tissues only [62]. However, in *B. napus* this gene specifically expressed in developing seed tissues [63] confirming our current observations. Moreover, this gene is unevenly found in plants i.e., *A. thaliana* genome has one copy compared to *B. napus*, *G.max*, *Z.mays*, *S. indicum*, and *G. hirsutum* whose genomes has multiple copies of the *FAD2* gene [64–69]. Variation in gene copies could cause differential expression patterns and one of the examples is highlighted in *G. max* where *GmFAD2.1* strongly and exclusively expressed in developing embryos and *GmFAD2.2*, and *GmFAD2.3* genes showed comparatively lower expression in all tissues [70].

The upstream regions of *FAD* and *FAB* genes are enriched in stress-related *cis*-elements [71]. Abiotic (heat) and diseases are major factors limiting oil seed production. In this regard, extensive studies have also been carried out to assess the expression pattern of FAD genes under multiple stresses including heat, drought, salinity, and cold stresses.  For instance, the *BolFAD2* and *BolFAD7* gene induced their expression under elevated temperatures. These genes have already been reported as crucial genes in plant adaptation [14, 22, 25, 72]. Moreover, the *FAD7* gene of *M. truncatula* also got up-regulated against higher temperatures validating our current observations [28]. Similarly, *FADs* were also found to be abundantly expressed against cold stress and production of polyunsaturated fatty acids is one of the most commons mechanism to combat cold stress/low temperature [73–76]. In addition to abiotic stress, we have also identified potential genes induced under *X. campestris* inoculum. Such investigation have also been carried out under different biotic stresses and in sunflower *HaFAD3.1* and *HaADS6* genes were found to be expressed at a higher rate under *Orobanche cumana* inoculum indicating that *FADs* have potential against multiple biotic stresses [53].

## Conclusions

*FADs* add unsaturated bonds to the hydrocarbon chain of fatty acids. Broadly FADs are categorized into two classes: soluble and non-soluble. These two classes share no sequence similarities at the sequence level and evolved independently.

Upstream regions of most *FAD* genes are enriched with stress-responsive *cis*-regulatory elements highlighting their potential in stress response and their presence is also influenced gene expression as well.

## Methods

### Data base research, identification, and physio-chemical properties of *FAD* like genes in *B. carinata*, and its progenitors

Genomes, GFF (General feature format), CDS (coding sequence), and peptide sequence files of *B. carinata*, *B. nigra*, and *B. oleracea* were retrieved from *Brassica* data base BRAD (http://brassicadb.cn). The *FAD* genes of *Arabidopsis* were downloaded from TAIR data base (https://www.arabidopsis.org) and used as the query sequences. TB tools v 1.09832 was used to identify the all-*FAD* like genes in three *Brassica* genomes through BLASTp program against a threshold level of E value < 1e-5. The retrieved protein sequences were subjected to CDD to select the sequences with conserved domains only [77]. Moreover, the physio-chemical properties of identified proteins like protein length, molecular weight, and *pI* were calculated using the Expasy platform (https://web.expasy.org/compute_pi/) [78].

### Multiple sequence alignment, phylogenetic and motif analysis

The retrieved protein of *Brassicas* along with *A. thaliana* and *O. sativa* were aligned in Clustal Omega (https://www.ebi.ac.uk/Tools/msa/clustalo/) [79] and MEGA X v 10.2.2 was used to construct an un-rooted neighbor-joining tree and adjusted the boost trap repeats to 1000- with JTT (Jones-Taylor-Thornton) + G (Gamma distributed) model [80]. Further, the retrieved protein sequences were submitted to the MEME suit server with default parameters to know the motif composition of the identified proteins.

### Promotor, gene structure, and gene divergence analysis

The 1500 bp upstream nucleotide sequence from the start position of each gene (ATG) was retrieved using TB tool FASTA extract and submitted to online Plant care (http://bioinformatics.psb.ugent.be/webtools/plantcare/html) data-base to identify the *cis*-regulatory elements in promotor regions [81].

Genes general information i.e., gene length, intron/exon distribution, and physical gene location were extracted from GFF files with the help of GFX select tool of TB tools [82]. Orthologues gene pairs were picked up from the adjacent nodes of the phylogenetic tree to estimate the gene divergence. The Ka and Ks values of orthologous gene pairs were calculated through TB tools. Moreover, the selection type was estimated using ka/ks ratio.

### Transcriptomic data analysis of *FAD* like genes under normal and stressed conditions

The ENA EBI (https://www.ebi.ac.uk/ena/browser/home) database was used to retrieve the transcriptomic data of different tissues under normal and stressed conditions. The transcriptomic data of embryo development stages and seed coat development stages was acquired from bio project PRJNA641876 and expression data of these tissues under different temperature regimes was acquired from PRJNA524852. Expression data of PRJNA524852 was used to examine the gene expression of root tissues under phosphate and zinc stress. Further, transcriptomic data under *X. campestris* was obtained from PRJNA421190. The data was processed through the Galaxy platform using sailfish alignment tool and TPM (Transcript per Million) values were isolated by putting the putative gene IDs. The data was normalized (log2) and presented with the Morpheus heat map tool (https://software.broadinstitute.org/morpheus/).

## Supplementary Information

**Additional file 1: Figure S1. **Phylogenetic distribution of *FAD* genes along with *A. thaliana* and *O. sativa.*

**Additional file 2: Figure S2. **Phylogenetic distribution of *FAB* genes along with *A. thaliana* and *O. sativa.*

**Additional file 3: Table S1. **Molecular and physio-chemical parameters of both *FAD* and *FAB* like genes of *Brassicas*

**Additional file 4: Table S2. ***Cis*-regulatory elements of *FAD* and *FAB* like genes and their expression in different tissues.

**Additional file 5: Table S3. **Gene divergence analysis of membrane bound *FAD* and *FAB* orthologous gene pairs.

**Additional file 6: Table S4. **Transcriptomic data of *FAD* and *FAB* like genes in diferent tissues under biotic and abiotic stresses. 

## Data Availability

Genomes of *Brassica* species were acquired from (http://brassicadb.cn ) and query sequences of *A. thaliana* were retrieved from (https://www.arabidopsis.org ) whereas FAD related proteins of *O. sativa* were obtained from (http://rice.uga.edu/). Online transcriptomic data (PRJNA524852, PRJNA641876, PRJNA524852, and PRJNA421190) of different tissues under normal and stressed conditions were acquired from (https://www.ebi.ac.uk/ena/browser/home).
