# Supplementary figures and images for: Comparative transcriptomic and evolutionary analysis of FAD-like genes of Brassica species revealed their role in fatty acid biosynthesis and stress tolerance
Source: BMC Plant Biol. 2023 May 12;23:250. doi: 10.1186/s12870-023-04232-9 (PMC10176799; doi:10.1186/s12870-023-04232-9)

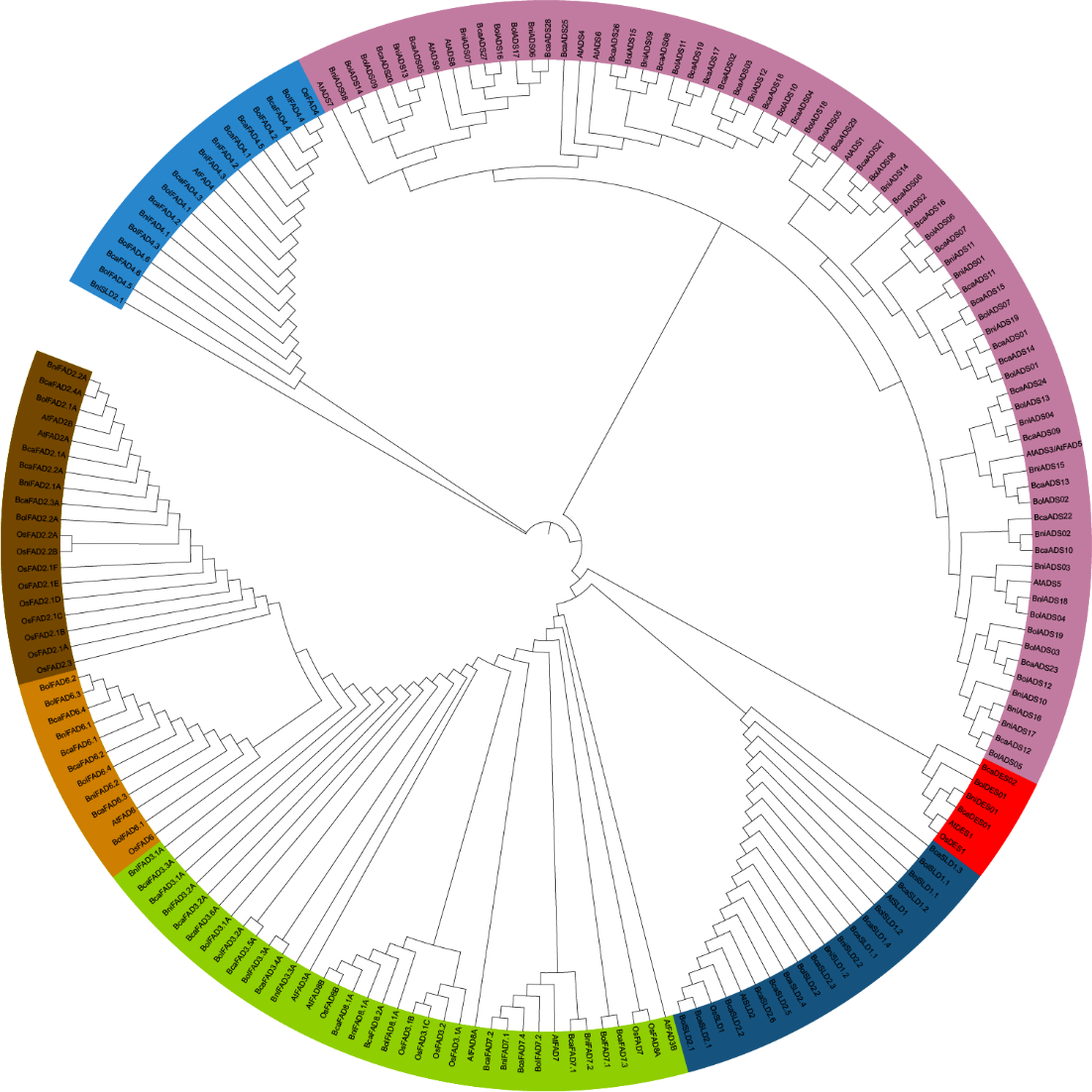


**Figure S1: Phylogenetic tree of membrane bound *FAD* genes along with *A. thaliana* and *O. sativa***

Supplement: Supplementary file 1 — Additional file 1: Figure S1. Phylogenetic distribution of FAD genes along with A. thaliana and O. sativa. [file 12870_2023_4232_MOESM1_ESM.docx]

**
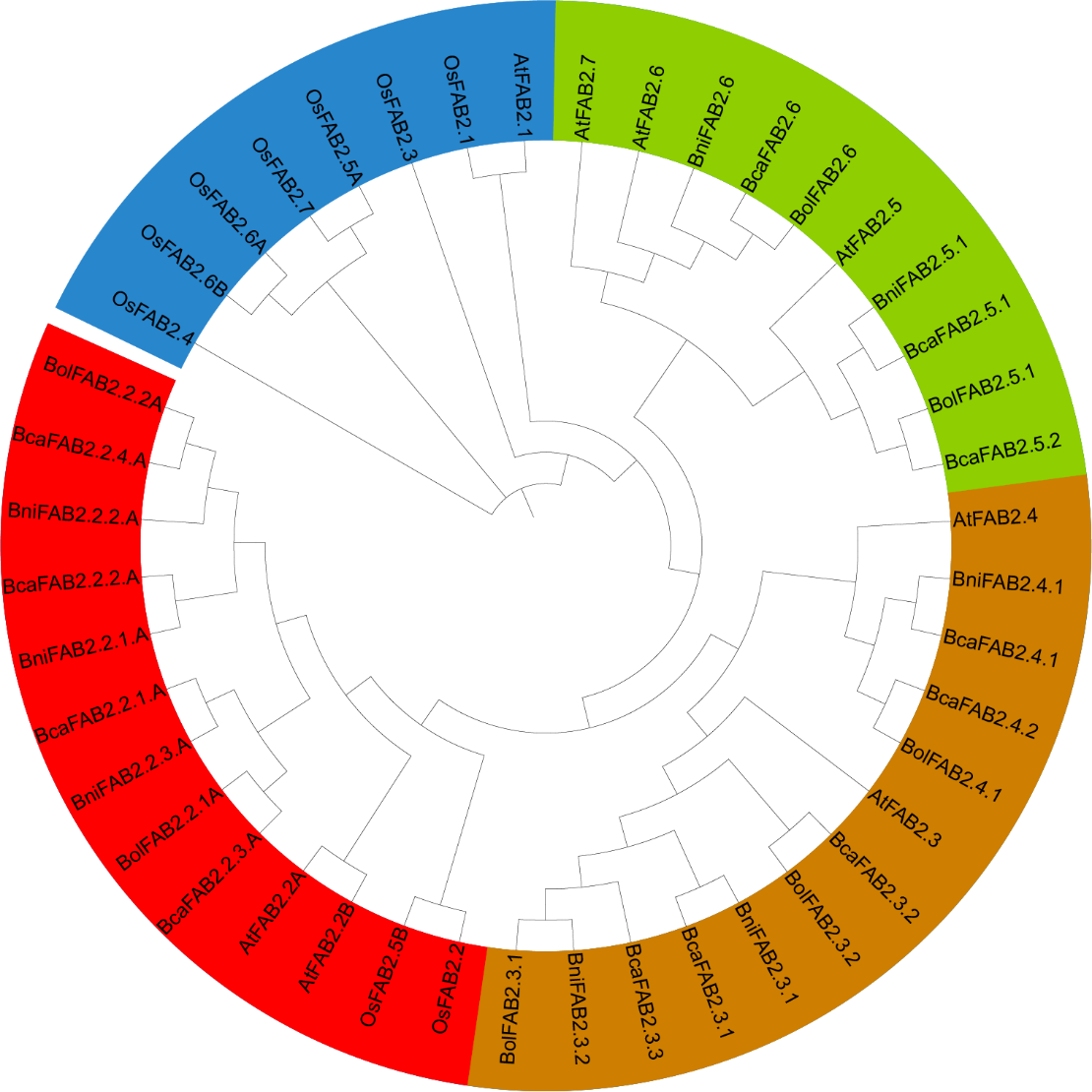
**

**Figure S2: Phylogenetic tree of *FAB* genes along with *A. thaliana* and *O. sativa***

Supplement: Supplementary file 2 — Additional file 2: Figure S2. Phylogenetic distribution of FAB genes along with A. thaliana and O. sativa. [file 12870_2023_4232_MOESM2_ESM.docx]
